# Supplementary material for: Ultrafast silicon photonic reservoir computing engine delivering over 200 TOPS
Source: Nat Commun. 2024 Dec 30;15:10841. doi: 10.1038/s41467-024-55172-3 (PMC11686264; doi:10.1038/s41467-024-55172-3)
Supplement: Supplementary file 1 — Supplementary Information [file 41467_2024_55172_MOESM1_ESM.pdf]

Supplementary Information: Ultrafast Silicon Photonic Reservoir Computing Engine Delivering Over 200 TOPS

DONGLIANG WANG, YIKUN NIE, GAOLEI HU, HON KI TSANG, CHAORAN HUANG*

Department of Electronic Engineering, The Chinese University of Hong Kong, Shatin, Hong Kong SAR, China.
*Corresponding author E-mail: crhuang@ee.cuhk.edu.hk

Supplementary Note 1. Principle of amplitude modulation
Supplementary Note 2. Device structure
Supplementary Note 3. Principle of all-optical NG-RC
Supplementary Note 4. Multi-wavelength operation experimental setup
Supplementary Note 5. Energy consumption
Supplementary Note 6. System stability
Supplementary Note 7. Scalability in spatial domain
Supplementary Note 8. Correlation between system performance and output quantity
Supplementary Note 9. Implementation of higher-order polynomials
Supplementary Note 10. Comparison table

Supplementary Note 1. PRINCIPLE OF AMPLITUDE MODULATION

In our experiment, we encode the signal in the amplitude domain of the light. The modulator we use is configured in a push-pull arrangement. When the modulator is biased at the null points, the optical field after modulation can be expressed as

$$E_{out} = E_{in} sin(\frac{\pi}{2}\frac{V}{V_\pi}) \quad \text{(S1)}$$

where E_{in} and E_{out} are the optical fields before and after the modulator, respectively. V is the voltage applied to the modulator. For $|V| < V_\pi/4$, the corresponding E_{out} can be approximated by a linear function written as

$$E_{out} = \frac{\pi}{2}\frac{E_{in}}{V_\pi} V \quad \text{(S2)}$$

where E_{out} is linearly proportional to V. Therefore, we use $|V| < V_\pi/4$ to linearly encode the input data. When voltages V and $-V$ are applied, the corresponding light intensities are identical, but the laser amplitudes are opposite in sign.

Supplementary Note 2. DEVICE STRUCTURE

Our photonic NG-RC chip is fabricated on a silicon-on-insulator (SOI) platform as depicted in Supplementary Fig. 1. The structure comprises a star coupler and several integrated delay lines. The star coupler, with an area of 200 μm × 200 μm, has nine input ports and forty-five output ports.

The silicon waveguides in our system are composed of three layers: a 2.2-μm-thick oxide (SiO_2) cladding layer, a 220-nm-thick silicon (Si) layer, and a 2-μm-thick buried thermal oxide (SiO_2) layer. The width of the silicon layer of delay lines is 500 nm in our design. The group index of these delay lines is approximately 4.24 at λ = 1550 nm by FDTD simulation. To achieve a delay of 16.7 ps in our design, the length difference of the neighboring delay lines is set as 1.18 mm. We employ a 13-ps width optical pulse to evaluate the delay time of different delay lines, as depicted in Supplementary Fig. 2a. Supplementary Fig. 2b shows the output optical pulses after the input tested optical pulse passes through our structure. The adjacent delay line realizes Δt = 16.7 ps.

Supplementary Note 3. PRICIPLE OF ALL-OPTICAL NG-RC

Single wavelength operation: Fig. 1c,d of the main text present our proposed photonic RC chip, designed according to the NG-RC framework. The input data **X** is encoded onto the amplitude of

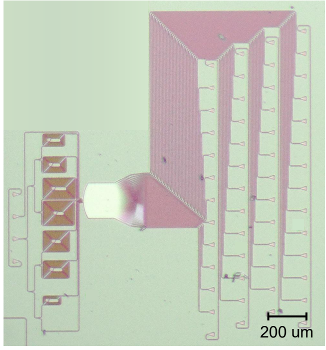

Supplementary Fig. 1. Device structure. The microscope photo of our photonic next-generation reservoir computing structure.

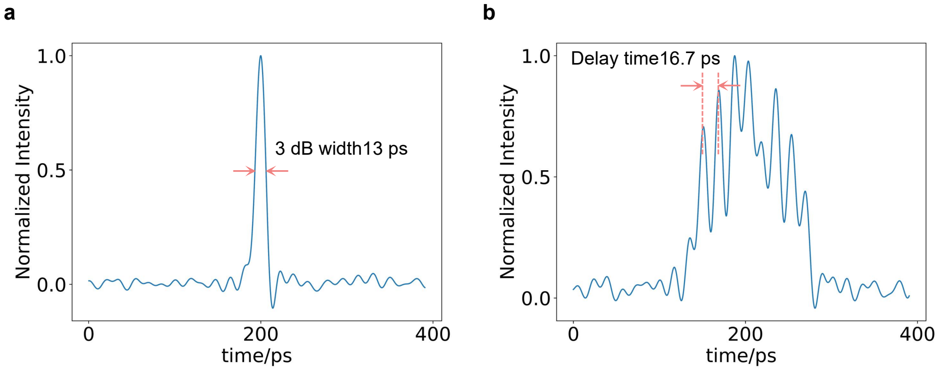

Supplementary Fig. 2. Delay line test. a The optical pulse used to test the delay time of our delay line. The 3 dB width of this pulse is 13 ps. **b** The output optical pulses after the input tested optical pulse passes through our structure. The neighboring delay line realizes a 16.7 ps delay.

a laser with wavelength λ. This input signal is then split into N delayed copies. These copies, along with an unmodulated laser representing the constant c, are fed into a star coupler. The adjacent delay lines introduce a time delay of Δt, corresponding to one symbol duration, ensuring that different time segments of the input data arrive simultaneously at the star coupler.

After passing through the star coupler, the output vector $\mathbf{y}_{star,\lambda}$ is expressed as

$$\mathbf{y}_{star,\lambda} = \boldsymbol{\omega}_{star,\lambda} \cdot (c \oplus \mathbf{X}), \tag{S3}$$

where $\boldsymbol{\omega}_{star,\lambda}$ is an $M \times (N+1)$ complex matrix, representing the transfer function of the star coupler at wavelength λ. Here, N denotes the number of delayed input copies and M is the dimension of the output vector.

The outputs from the star coupler are processed by an optical readout layer, implemented via an array of programmable MRRs. The resonance wavelengths of the MRRs are aligned with the input wavelengths. By adjusting the resonance through embedded thermal phase shifters, the fraction of light directed to the drop ports can be finely controlled. The phase shifter in the MRR tunes the amplitude of the signal, while a second phase shifter on the bus waveguide adjusts the signal's phase. This dual control enables precise manipulation of complex weights.

Then the output of the i_{th} output port after MRR is given by:

$$\begin{aligned} y_{MRR,i,\lambda} &= \omega_{MRR,i} y_{star,i,\lambda} \\ &= \omega_{MRR,i} \left(c_i + \sum_{n=1}^{N} a_{i,n} x_{t+1-n} \right) \end{aligned} \tag{S4}$$

where $\omega_{MRR,i}$ is a tunable complex weight produced by the i_{th} MRR, and c_i, and $a_{i,n}$ are constants, which are determined by the structure of the star coupler.

The outputs from the first $\frac{M}{2}$ rows of MRRs are combined and detected by one photodiode. The output of the photodiode can be written as:

$$\begin{aligned} y_{PD}^{+} &= \left| \sum_{i=1}^{M/2} y_{MRR,i,\lambda} \right|^2 = \left| \boldsymbol{\omega}_{\mathrm{MRR}}^{+} \mathbf{y}_{\mathrm{star},\lambda}^{+} \right|^2 \\ &= c^{+} + \sum_{n=1}^{N} \omega_{lin,n}^{+} x_{t+1-n} + \sum_{m=1}^{N} \sum_{n=m}^{N} \omega_{nonlinear,mn}^{+} x_{t+1-m} x_{t+1-n} \end{aligned} \tag{S5}$$

where $\boldsymbol{\omega}_{\mathrm{MRR}}^{+}$ is a complex weight vector given by the half rows of MRRs, and $\mathbf{y}_{star,\lambda}^{+}$ is the output vector from the first half output ports of star coupler. Here, c^{+}, $\omega_{lin,n}^{+}$, and $\omega_{nonlinear,mn}^{+}$ are determined by the star coupler and MRRs together.

By tuning the resonance of the MRRs and adjusting the phase using phase shifters, these coefficients, c^{+}, $\omega_{lin,n}^{+}$, and $\omega_{nonlinear,mn}^{+}$, can be controlled. However, due to the squaring effect of the photodetector, some coefficients are constrained to the range (0,1). To overcome this limitation and extend the coefficient range to (-1,1), we employ a balanced photodetector (BPD). The outputs from the second set of $\frac{M}{2}$ MRRs are combined and sent to a second photodiode. The differential signal between the two photodiodes is then expressed as

$$\begin{aligned} y_{BPD,\lambda} &= \left| \boldsymbol{\omega}_{\mathrm{MRR}}^{+} \mathbf{y}_{\mathrm{star},\lambda}^{+} \right|^2 - \left| \boldsymbol{\omega}_{\mathrm{MRR}}^{-} \mathbf{y}_{\mathrm{star},\lambda}^{-} \right|^2 \\ &= c^{+} - c^{-} + \sum_{n=1}^{N} (\omega_{lin,n}^{+} - \omega_{lin,n}^{-}) x_{t+1-n} + \sum_{m=1}^{N} \sum_{n=m}^{N} (\omega_{nonlinear,mn}^{+} - \omega_{nonlinear,mn}^{-}) x_{t+1-m} x_{t+1-n} \\ &= \underbrace{c}_{\text{constant}} + \underbrace{\sum_{n=1}^{N} \omega_{\mathrm{lin},n} x_{t+1-n}}_{\text{linear terms}} + \underbrace{\sum_{m=1}^{N} \sum_{n=m}^{N} \omega_{\mathrm{nonlinear},mn} x_{t+1-m} x_{t+1-n}}_{\text{quadratic terms}} \end{aligned} \tag{S6}$$

where $\boldsymbol{\omega}_{\mathrm{MRR}}^{+}$ and $\boldsymbol{\omega}_{\mathrm{MRR}}^{-}$ are complex weight vectors given by the first half and second half rows of MRRs, respectively. Here, $\mathbf{y}_{star,\lambda}^{+}$ and $\mathbf{y}_{star,\lambda}^{-}$ are the output vectors from the first half and the second half output ports of star coupler, respectively. This equation shows that the BPD output is a regression of the constant, linear, and quadratic terms of the inputs, with the coefficients c, ω_{lin}, and $\omega_{\mathrm{nonlinear}}$ programmable from -1 to 1 using the MRR array.

In digital NG-RC, for an input size of $N+1$, the number of tunable weights will be $\frac{(N+1)(N+2)}{2}$. This includes one weight for the constant, N weights for input terms, and $\frac{N(N+1)}{2}$ weights for the quadratic polynomials of inputs. In our photonic NG-RC, the number of output ports M of the star coupler determines the number of tunable parameters in the system. Therefore, the computing performance improves as the number of output ports M of the star coupler increases, and this performance reaches saturation when $M = \frac{(N+1)(N+2)}{2}$. Thus, the optical engine is functionally equivalent to the NG-RC when $M \geq \frac{(N+1)(N+2)}{2}$.

multi-wavelength operation: Due to the star coupler's wavelength-dependent response, we can enhance the system's scalability by utilizing wavelength-division multiplexing (WDM). As shown in Fig. 1c,d of the main text, the input data X is encoded onto an array of WDM lasers, each operating at one of P distinct wavelengths. At the output of the star coupler, signals corresponding to each wavelength are processed individually and weighted by separate columns of MRRs. Each column is designed with a distinct resonance wavelength, aligned with the corresponding WDM laser, allowing precise control and weighting of the optical signals across multiple wavelengths.

At the detection stage, signals from different wavelengths are combined linearly in the optical intensity domain. As a result, the output after the BPD is expressed as

$$y_{BPD} = \sum_{i=1}^{P} y_{BPD,\lambda_i} \tag{S7}$$

By employing WDM, the dimensionality of the output vector increases from M to $M \times P$, significantly expanding the system's computational capacity. The optical engine becomes functionally equivalent to the NG-RC when the number of tunable parameters satisfies $M \times P \geq \frac{(N+1)(N+2)}{2}$.

Supplementary Note 4. MULTI-WAVELENGTH OPERATION EXPERIMENTAL SETUP

Supplementary Fig. 3 illustrates the schematic of the multi-wavelength experimental setup. Light of different wavelengths is combined using a wavelength division multiplexer and then split into two equal branches by a 50/50 coupler. One branch is coupled into the photonic chip as a constant reference c, while the other is modulated by a thin-film lithium niobate intensity modulator.

The input signal, generated by an arbitrary waveform generator, is encoded onto the amplitude of the light by the modulator. Here, the light of different wavelengths carries the same signal. The modulated light is then amplified by an erbium-doped fiber amplifier (EDFA) before being injected into the photonic chip. The optical outputs are coupled out and the light of different wavelengths is split by optical wavelength division demultiplexer. The light is detected using an off-the-shelf photodetector and digitized by a real-time oscilloscope. Finally, the readout layer is trained using a digital computer.

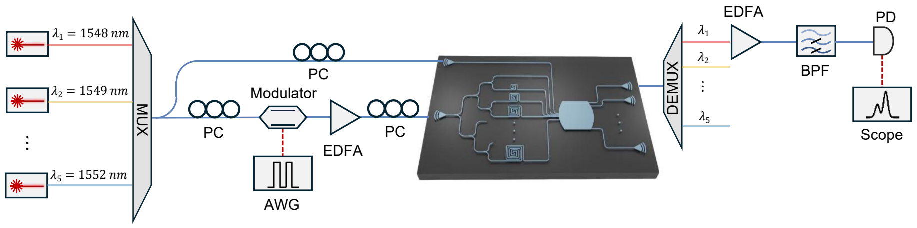

Supplementary Fig. 3. Schematic of multi-wavelength experimental setup. MUX, wavelength division multiplexer. PC, polarization controller. AWG, arbitrary waveform generator. EDFA, erbium-doped fiber amplifier. BPF, bandpass filter. DEMUX, wavelength division demultiplexer. PD, photodiode.

Supplementary Note 5. ENERGY CONSUMPTION

Our all-optical engine system consists of a laser, an erbium-doped fiber amplifier (EDFA), a 256 GSamples/s digital-to-analog converter (DAC), an on-chip modulator, ninety metal heaters, and an on-chip balanced photodetector paired with a 256 GSamples/s analog-to-digital converter (ADC). The power consumption of each component is estimated as follows.

(1) The laser used in our experiment is a PPCL500 with a power consumption of approximately 1W.

(2) The energy consumption of EDFA is estimated using the formula $\frac{\lambda_S}{\lambda_p}(P^s_{out} - P^s_{in})/\eta$ [1], where the signal wavelength is $\lambda_s = 1550nm$, the pump wavelength is $\lambda_p = 1480nm$, the output power is $P^s_{out} \approx 20dBm$, the input power is $P^s_{in} \approx 0dBm$, and the wall- plug efficiency is $\eta \approx 0.3$. Based on these parameters, the energy consumption of EDFA is approximately 0.35 W.

(3) For optical signal generation, the DAC-based transmitter in [2] achieves an energy efficiency of 2.8 pJ per Baud, while the modulator achieves an energy efficiency of 42 fJ per Baud [3]. Additionally, the modulator bias consumes approximately 25 mW. Therefore, the combined energy consumption of the DAC (operating at a sampling rate of 256 GSamples/s) and the modulator is approximately 0.75 W.

(4) The metal heaters in our system are used for phase shifting. Based on our experimental measurements, the power consumption of the metal heater phase shifter for a 2π phase shift is approximately 50 mW. We assume that an average π phase shift for each of the ninety metal heaters. Thus, the total energy consumption of the ninety metal heaters is approximately 2.25 W.

(5) For optical signal detection, the ADC-based receiver achieves an energy efficiency of 2.82 pJ per Baud [4], while the photodetector achieves an energy efficiency in the range of a few fJ per bit [5]. Therefore, the total energy consumption of the ADC (operating at a sampling rate of 256 GSamples/s) and the photodetector is approximately 0.75 W.

In summary, combining the energy consumption of all components—laser, EDFA, DAC, modulator, metal heaters, ADC, and photodetector—the total energy consumption of the all-optical engine is approximately 5.1 W.

In single-wavelength operation, our all-optical engine can be viewed as performing three key operations: an $(N+1) \times M$ complex matrix operation (via the star coupler), two $\frac{M}{2} \times 1$ complex matrix operations (through the microring weight banks), and two square operations (in the

balanced photodetector), where N is the number of delayed input copies and M is the number of outputs.

As noted in [6, 7], each complex multiplication involves 4 real multiplications and 2 real summations, while each complex summation involves 2 real summations. Thus, the $(N+1) \times M$ complex matrix operation involves $(N+1)M$ complex multiplications and NM complex summations, resulting in a total of $6(N+1)M + 2NM$ operations. Similarly, two $\frac{M}{2} \times 1$ complex matrix operations consist of M complex multiplications and $M-2$ complex summations, leading to $6M + 2(M-2)$ computational operations. Finally, the two complex square operations contribute an additional 12 operations.

As a result, the optical engine demonstrated in our experiment operates at a line rate of 60 Gbaud and achieves a computational throughput of 211 Tera operations per second (TOPS), when $N = 8$ and $M = 45$. The system's energy efficiency is approximately 41 TOPS/W with these components. For comparison, NVIDIA's H100 GPU has an energy efficiency of roughly 0.15 TOPS/W [6]. Notably, as illustrated in Supplementary Fig. 4, when the reservoir size increases, our optical engine demonstrates even greater energy efficiency advantages over GPU H100, especially when processing large-scale NGRC tasks.

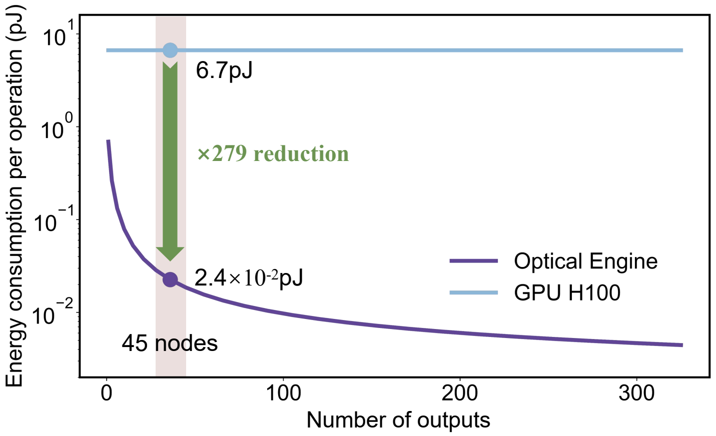

Supplementary Fig. 4. Energy consumption. The energy consumption per operation of NG-RC with different numbers of outputs completed by our all-optical engine (as shown in Fig. 1c of the main text) and GPU H100.

Supplementary Note 6. SYSTEM STABILITY

In our experiment, we introduced the signal and constant inputs into the on-chip photonic NG-RC through two separate optical fibers. Since the signal path has 8 delayed input copies, we set the optical power ratio between the signal and constant ports to approximately 8:1 during the experiment. However, due to the phase instability in the two fiber paths, phase variance is introduced.

We conduct an experiment to investigate whether the phase variance can affect the system performance, as shown in Supplementary Fig. 5. In our experiment, we injected unmodulated light into the signal and constant input ports by two optical fibers with an optical power ratio of 8:1. The temporal variation in optical power at one of the star coupler's outputs was measured, as shown by the yellow lines in Supplementary Fig. 5. Based on our measurements, the phase fluctuation occurs at a frequency on the order of KHz, as shown in the upper figure in Supplementary Fig. 5. During our experiments, each measurement cycle was completed within 2 microseconds. As shown in the lower figure in Supplementary Fig. 5, the output power remained consistent within each measurement cycle. The experimental results confirm that phase variance has a negligible impact on computing accuracy, as it occurs on a much slower timescale than the computing output.

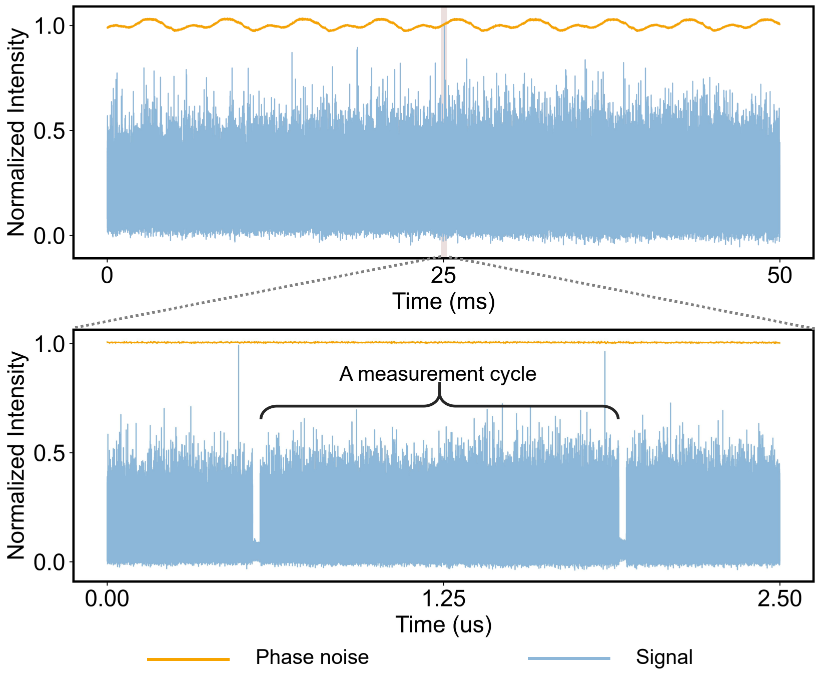

Supplementary Fig. 5. Environmental stability. Experimental demonstration of environmental stability. The blue lines represent the experimental data during task execution, while the yellow lines correspond to the results from measuring environmental stability.

Supplementary Note 7. SCALABILITY IN SPATIAL DOMAIN

Due to the simplicity of our system architecture and the small size of each component, our design exhibits excellent spatial scalability. The primary limiting factor for our system is the signal-to-noise ratio (SNR). To maintain a reasonable SNR, the minimum output power should be approximately -20 dBm, while the photonic chip can handle a maximum input power of around 20 dBm. Under ideal conditions, with no significant losses, a star coupler could theoretically distribute light evenly across up to 10,000 spatial nodes. However, accounting for typical losses in the delay lines and star coupler (around 3 dBm), the system can realistically support over 5,000 spatial nodes.

The star coupler must have a minimum width of 2.5 mm to accommodate 5,000 waveguides, each 500 nm wide, at the output. Therefore, the area of this star coupler is estimated to be 2.5 mm $\times$ 2.5 mm = 6.25 mm^2. This compact design ensures scalability while maintaining the necessary performance metrics for large-scale implementations.

Supplementary Note 8. CORRELATION BETWEEN SYSTEM PERFORMANCE AND OUTPUT QUANTITY

In the RC system, the number of outputs is directly linked to its computational capacity.

Fixed input dimension: In our experiment, the input dimension is 9, leading to an output comprising a constant term, 8 linear terms, and 36 quadratic terms, totaling 45 degrees of freedom. To fully control the coefficients of these 45 degrees of freedom, we require 45 distinct outputs.

With the input dimension fixed at 9, we evaluate the system's performance as a function of the number of output ports using the NARMA10 task, as illustrated in Supplementary Fig. 6. When the number of outputs is below 45 (as indicated in the experiment part of Supplementary Fig. 6), the coefficients corresponding to the 45 degrees of freedom can not be fully optimized. As the number of outputs increases, more coefficients can be adjusted, resulting in a reduction in NMSE. However, once the number of outputs exceeds 45 (as shown in the simulation part of Supplementary Fig. 6), we can only adjust the coefficients of 45 degrees of freedom, leading to saturation and stabilization in NMSE.

Varying input dimension: When we increase the input port number, which is $N+1$ (N signals

and a constant), and the number of distinct outputs accordingly changes to $\frac{(N+1)(N+2)}{2}$, we can find the NMSE decreases exponentially with the number of outputs in the Supplementary Fig. 7. In Supplementary Fig. 7, we also compare our system's performance with other implementations of the same task [8–11]. The results underscore a key advantage of our approach: it achieves significantly lower NMSE with fewer feature vectors, thereby reducing both energy consumption and the chip's physical footprint.

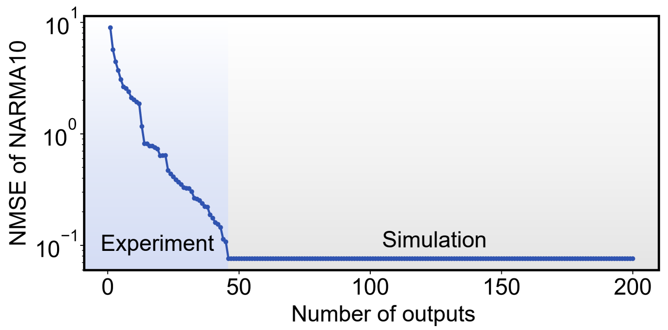

Supplementary Fig. 6. NMSE of NARMA10 task with an increasing number of outputs, when the input dimension is fixed at 9.

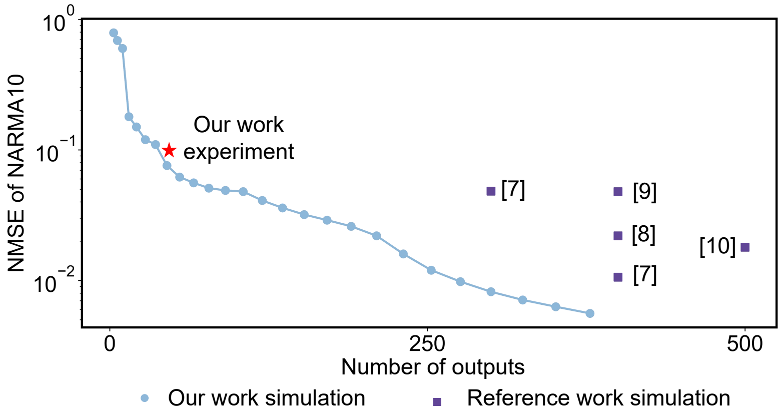

Supplementary Fig. 7. NMSE of NARMA10 task with an increasing number of outputs, when the input dimension changes accordingly with the number of outputs.

Supplementary Note 9. IMPLEMENTATION OF HIGHER-ORDER POLYNOMIALS

While a simple quadratic polynomial offers good computational power, certain problems may require higher-order polynomials. Our system can implement these higher-order polynomials by cascading modulators at the input stage. As illustrated in Supplementary Fig. 8, at the input stage, unmodulated light is split into three paths on-chip. In the first path, the light remains unmodulated. In the second path, the data X is encoded onto the light's amplitude using a modulator. In the third path, the data X^2 is encoded onto the light's amplitude using two cascaded modulators. The inputs X and X^2 are both split into N delayed copies. These copies, along with an unmodulated laser representing the constant c, are sent into a star coupler.

After passing through the star coupler, the output vector $\mathbf{y}_{star}$ is expressed as:

$$\mathbf{y}_{star} = \boldsymbol{\omega}_{star} \cdot (c \oplus \mathbf{X} \oplus \mathbf{X^2}) \tag{S8}$$

where $\boldsymbol{\omega}_{star}$ is an $M \times (2N+1)$ complex matrix, representing the transfer function of the star coupler. Here, N denotes the number of delayed input copies and M is the dimension of the output vector.

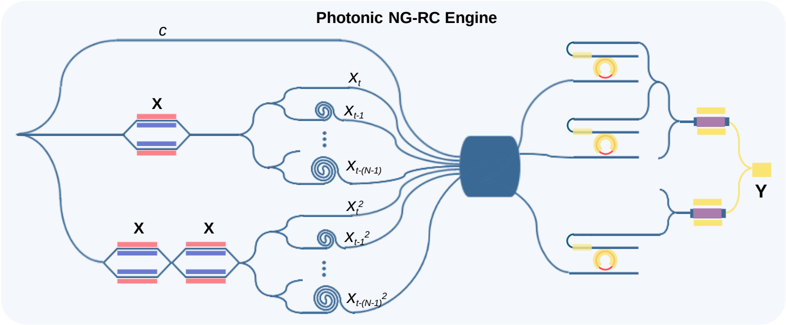

Supplementary Fig. 8. Implementation of higher-order polynomials. Photonic reservoir computing framework for generating higher-order terms.

The star coupler outputs are processed by an optical readout layer composed of a programmable MRR array. By tuning the resonance with integrated thermal phase shifters, the amount of light routed to the drop ports is precisely controlled. The MRR phase shifter modulates the signal's amplitude, while a second phase shifter on the bus waveguide adjusts its phase. This combination allows for accurate control of complex weights.

Then the output of the i_{th} output port after MRR is given by:

$$\begin{aligned} y_{MRR,i} &= \omega_{MRR,i} y_{star,i} \\ &= \omega_{MRR,i}\left(c_i + \sum_{n=1}^{N} a_{i,n}x_{t+1-n} + \sum_{n=1}^{N} b_{i,n}x^2_{t+1-n}\right) \end{aligned} \tag{S9}$$

where $\omega_{MRR,i}$ is a tunable complex weight by the i_{th} MRR, and c_i, $a_{i,n}$, and $b_{i,n}$ are constants, which are determined by the structure of the star coupler.

The outputs from the first $\frac{M}{2}$ rows of MRRs are combined and detected by one photodiode. The output of the photodiode can be written as:

$$\begin{aligned} y^+_{PD} &= \left|\sum_{i=1}^{M/2} y_{MRR,i}\right|^2 = \left|\boldsymbol{\omega}^+_{\text{MRR}}\mathbf{y}^+_{\text{star}}\right|^2 \\ &= c^+ + \sum_{n=1}^{N}\omega^+_{lin,n}x_{t+1-n} + \sum_{m=1}^{N}\sum_{n=m}^{N}\omega^+_{quadratic,mn}x_{t+1-m}x_{t+1-n} \\ &+ \sum_{m=1}^{N}\sum_{n=m}^{N}\omega^+_{cubic,mn}x_{t+1-m}x^2_{t+1-n} + \sum_{m=1}^{N}\sum_{n=m}^{N}\omega^+_{quartic,mn}x^2_{t+1-m}x^2_{t+1-n} \end{aligned} \tag{S10}$$

where $\boldsymbol{\omega}^+_{\text{MRR}}$ is a complex weight vector given by the first half rows of MRRs, and $\mathbf{y}^+_{star}$ is the output vector from the first half output ports of star coupler. Here, c^+, $\omega^+_{lin,n}$, $\omega^+_{quadratic,mn}$,$\omega^+_{cubic,mn}$, and $\omega^+_{quartic,mn}$ are determined by the star coupler and MRRs together.

By tuning the resonance of the MRRs and adjusting the phase using phase shifters, these coefficients can be controlled. The outputs from the second set of $\frac{M}{2}$ MRRs are combined and sent to a second photodiode. The differential signal between the two photodiodes is then expressed as:

$$\begin{aligned} y_{BPD} &= \left|\boldsymbol{\omega}^+_{\text{MRR}}\mathbf{y}^+_{\text{star},\lambda}\right|^2 - \left|\boldsymbol{\omega}^-_{\text{MRR}}\mathbf{y}^-_{\text{star},\lambda}\right|^2 \\ &= \underbrace{c}_{\text{constant}} + \underbrace{\sum_{n=1}^{N}\omega_{\text{lin},n}x_{t+1-n}}_{\text{linear terms}} + \underbrace{\sum_{m=1}^{N}\sum_{n=m}^{N}\omega_{\text{quadratic},mn}x_{t+1-m}x_{t+1-n}}_{\text{quadratic terms}} \\ &+ \underbrace{\sum_{m=1}^{N}\sum_{n=m}^{N}\omega_{\text{cubic},mn}x_{t+1-m}x^2_{t+1-n}}_{\text{cubic terms}} + \underbrace{\sum_{m=1}^{N}\sum_{n=m}^{N}\omega_{\text{quartic},mn}x^2_{t+1-m}x^2_{t+1-n}}_{\text{quartic terms}} \end{aligned} \tag{S11}$$

where $\boldsymbol{\omega}_{\text{MRR}}^{+}$ and $\boldsymbol{\omega}_{\text{MRR}}^{-}$ are complex weight vectors given by the first half and the second half rows of MRRs, respectively. Here, $\mathbf{y}_{star}^{+}$ and $\mathbf{y}_{star}^{-}$ are the output vectors from the first half and second half output ports of the star coupler, respectively. This equation shows that the BPD output is a regression of the constant, linear, quadratic, cubic, and quartic terms of the inputs, with the coefficients c, ω_{lin}, $\omega_{\text{quadratic}}$, ω_{cubic}, and ω_{quartic}, programmable from -1 to 1 using the MRR array.

Supplementary Note 10. COMPARISON TABLE

To further illustrate the advancement of our system, we provide a detailed comparison with classic photonic RC systems [8, 12–21]. This comparison covers components, operation speed, scalability, and task performance. The table demonstrates that our system achieves the highest processing speed and computing performance while using minimal resources, including chip area, power consumption, and output port number.

Table S1 Performance comparison with previous photonic reservoir computing systems. When achieving the same results as the reference works, the required node count, corresponding chip area, and energy consumption in our work are highlighted in red.

Systems	Components required for reservoir layer	Operation Speed[1] (GHz)	Chip Area Per Node[2]	Effective Node No. Per Area in Unit Time[3]	Task Performance[4]			
					Task	Node Count[5]	Chip Area[6]	Energy Consumption[7]
	Our work							
This work	**On-chip delay lines, star coupler.**	**60**	**0.04 mm^2**	**4.7×10^8 (/1 cm^2/1 μ s)**	See details below<br>Other work<br>/**Our work**			
	Conventional RC							
Vandoorne et al. [11]	On chip delay lines and MMIs.	0.125~12.5	1 mm^2	1.3×10^6 (/1 cm^2/1 μ s)	XOR BER = 0	11 **/ 6**	16 mm^2 **/0.32 mm^2**	1.7 nJ (digital readout) 1.4 nJ (optical readout) **/53 pJ (optical readout)**
Vinckier et al. [7]	Fiber delay line, couplers, attenuator, piezoelectric fiber stretcher,	9×10^{-4}	N/A	45 (/1 μ s)	NARMA10 NMSE = 0.046	300 **/78**	N/A **/1.55 mm^2**	1.3 uJ (digital readout) 1.4 uJ (optical readout) **/113 pJ (optical readout)**
Larger et al. [12]	Phase modulator, circulator, fiber delay line, PD, low pass filter, RF driver.	1.6×10^{-2}	N/A	5.9×10^3 (/1 μ s)	Spoken digital recognition TI46 WER ≈ 0	371 **/378**	N/A **/5.7 mm^2**	87 nJ (digital readout) 87 nJ (optical readout) **/364 pJ (optical readout)**
Rafayelyan et al. [13]	Scattering medium, collimator lens system, expander lens system	4×10^{-9}	N/A	0.33 (/1 cm^2/1 μ s)	Kuramoto-Sivashinsky time series NRMSE = 0.298	50000 **/2500**	60 mm^2 **/33 mm^2**	3.6 J (digital readout) **/2.1 nJ (optical readout)**
Sunada et al. [14]	On-chip multimode waveguide	12.5	0.06 mm^2	8.1×10^7 (/1 cm^2/1 μ s)	Santa Fe NMSE = 0.039	270 **/28**	4 mm^2 **/0.75 mm^2**	4.5 uJ (digital readout) **/71 pJ (optical readout)**

Nakajima et al. [15]	MZI mesh, on-chip delay lines, coherent cavities, variable optical attenuator,	1.9	41 mm^2	7.3×10^4 (/1 cm^2/1 μ s)	Santa Fe NMSE = 0.06	256 **/15**	658 mm^2 **/0.51 mm^2**	6.8 nJ (digital readout) 3.7 nJ (optical readout) **/60 pJ (optical readout)**
Lupo et al. [16]	EDFA, phase modulator, RF source, RF amplifier, fiber delay lines, fiber couplers, programmable spectral filter, PD, MZM modulator	0.01	N/A	2.8×10^2 (/1 μ s)	nonlinear channel equation (24 dB) SER = 10^{-4}	40 **/45**	N/A **/1 mm^2**	350 nJ (optical readout) **/85 pJ (optical readout)**
Shen et al. [17]	Fiber couplers, circulators, fiber delay lines, attenuators, slave lasers, EDFAs, polarization controllers	0.25	N/A	8×10^4 (/1 μ s)	25 Gbps OOK 50km BER =10^{-3}	240 **/105**	N/A **/1.95 mm^2**	14 nJ (digital readout) 14 nJ (optical readout) **/135 pJ (optical readout)**
Antonik et al. [18]	Collimator lens system and expander lens system	2×10^{-9}	N/A	0.054 (/1 cm^2/1 μ s)	N/A			
	**Other photonic NG-RC**							
Wang et al. [19]	ground glass diffuser, collimator lens system, expander lens system	1×10^{-8}	N/A	7.3×10^{-4} (/1 cm^2/1 μ s)	Kuramoto-Sivashinsky time series NRMSE = 0.298	2500	380 mm^2 **/33 mm^2**	1.45 J **/2.13 nJ (optical readout)**
Cox et al. [20]	Amplitude modulator, fiber circulator,	1.7×10^{-4}	N/A	1×10^3 (/1 μ s)	Lorenz 63 NRMSEy = 1.23×10^{-2} NRMSEz = 1.89×10^{-2}	1000	N/A **/13.9 mm^2**	9.5 uJ (digital readout) 10.4 uJ (optical readout) **/883 pJ (optical readout)**

[1]Operation speed means the prediction, emulation, or classification speed in photonic reservoir computing.

[2]Chip area only includes the area of input ports and the reservoir layer area but excludes the output layer, as it is a standard linear regression layer and most of work is done offline on a computer.

[3]Number of reservoir computing nodes achievable within a 1 square centimeter space and a 1 microsecond time frame.

[4]For a specific task, our system achieves the same simulation results as the reference works. The parameters of our optical engine are highlighted in red.

[5]Number of reservoir computing nodes required to complete a specific task.

[6]Area required for reservoir computing to complete a specific task.

[7]Theoretical energy consumed to perform a single prediction or classification.

SUPPLEMENTARY REFERENCES

1. H. Feng, T. Ge, X. Guo, *et al.*, "Integrated lithium niobate microwave photonic processing engine," Nature **627**, 80–87 (2024).
2. M. A. Kossel, V. Khatri, M. Braendli, *et al.*, "8.3 an 8b dac-based sst tx using metal gate resistors with 1.4 pj/b efficiency at 112gb/s pam-4 and 8-tap ffe in 7nm cmos," in *2021 IEEE International Solid-State Circuits Conference (ISSCC)*, vol. 64 (IEEE, 2021), pp. 130–132.
3. C. Wang, M. Zhang, X. Chen, *et al.*, "Integrated lithium niobate electro-optic modulators operating at cmos-compatible voltages," Nature **562**, 101–104 (2018).
4. A. Khairi, Y. Krupnik, A. Laufer, *et al.*, "A 1.41-pj/b 224-gb/s pam4 6-bit adc-based serdes receiver with hybrid afe capable of supporting long reach channels," IEEE J. Solid-State Circuits **58**, 8–18 (2022).
5. D. Benedikovic, L. Virot, G. Aubin, *et al.*, "Silicon-germanium avalanche receivers with fj/bit energy consumption," IEEE J. Sel. Top. Quantum Electron. **28**, 1–8 (2021).
6. Z. Xu, T. Zhou, M. Ma, *et al.*, "Large-scale photonic chiplet taichi empowers 160-tops/w artificial general intelligence," Science **384**, 202–209 (2024).
7. T. Zhou, X. Lin, J. Wu, *et al.*, "Large-scale neuromorphic optoelectronic computing with a reconfigurable diffractive processing unit," Nat. Photonics **15**, 367–373 (2021).
8. Q. Vinckier, F. Duport, A. Smerieri, *et al.*, "High-performance photonic reservoir computer based on a coherently driven passive cavity," Optica **2**, 438–446 (2015).
9. X. Liang, Y. Zhong, J. Tang, *et al.*, "Rotating neurons for all-analog implementation of cyclic reservoir computing," Nat. communications **13**, 1549 (2022).
10. L. Appeltant, M. C. Soriano, G. Van der Sande, *et al.*, "Information processing using a single dynamical node as complex system," Nat. communications **2**, 468 (2011).
11. L. Appeltant, G. Van der Sande, J. Danckaert, and I. Fischer, "Constructing optimized binary masks for reservoir computing with delay systems," Sci. reports **4**, 3629 (2014).
12. K. Vandoorne, P. Mechet, T. Van Vaerenbergh, *et al.*, "Experimental demonstration of reservoir computing on a silicon photonics chip," Nat. communications **5**, 3541 (2014).
13. L. Larger, A. Baylón-Fuentes, R. Martinenghi, *et al.*, "High-speed photonic reservoir computing using a time-delay-based architecture: Million words per second classification," Phys. Rev. X **7**, 011015 (2017).
14. M. Rafayelyan, J. Dong, Y. Tan, *et al.*, "Large-scale optical reservoir computing for spatiotemporal chaotic systems prediction," Phys. Rev. X **10**, 041037 (2020).
15. S. Sunada and A. Uchida, "Photonic neural field on a silicon chip: large-scale, high-speed neuro-inspired computing and sensing," Optica **8**, 1388–1396 (2021).
16. M. Nakajima, K. Tanaka, and T. Hashimoto, "Scalable reservoir computing on coherent linear photonic processor," Commun. Phys. **4**, 20 (2021).
17. A. Lupo, E. Picco, M. Zajnulina, and S. Massar, "Deep photonic reservoir computer based on frequency multiplexing with fully analog connection between layers," Optica **10**, 1478–1485 (2023).
18. Y.-W. Shen, R.-Q. Li, G.-T. Liu, *et al.*, "Deep photonic reservoir computing recurrent network," Optica **10**, 1745–1751 (2023).
19. P. Antonik, N. Marsal, D. Brunner, and D. Rontani, "Human action recognition with a large-scale brain-inspired photonic computer," Nat. Mach. Intell. **1**, 530–537 (2019).
20. H. Wang, J. Hu, Y. Baek, *et al.*, "Optical next generation reservoir computing," arXiv preprint arXiv:2404.07857 (2024).
21. N. Cox, J. Murray, J. Hart, and B. Redding, "Photonic next-generation reservoir computer based on distributed feedback in optical fiber," arXiv preprint arXiv:2404.07116 (2024).
